# UWB Circular Fractal Antenna with High Gain for Telecommunication Applications

**DOI:** 10.3390/s23084172

**Published:** 2023-04-21

**Authors:** Ibrahime Hassan Nejdi, Seddik Bri, Mohamed Marzouk, Sarosh Ahmad, Youssef Rhazi, Mustapha Ait Lafkih, Yawar Ali Sheikh, Adnan Ghaffar, Mousa Hussein

**Affiliations:** 1 Automatic and Energy Conversion (AEC) Faculty of Sciences and Technology, BP 523, Beni-Mellal 23000, Morocco; 2 Material and Instrumentations Group, Electrical Engineering Department, ESTM Moulay Ismail University, BP 3103, Meknes 50040, Morocco; 3 Microelectronics, Embedded Systems and Telecommunications (MiSET) Faculty of Sciences and Technology, BP 523, Beni-Mellal 23000, Morocco; 4 Department of Electrical Engineering and Technology, Government College University Faisalabad (GCUF), Faisalabad 38000, Pakistan; 5 Department of Signal Theory and Communications, Universidad Carlos III de Madrid (UC3M), 28911 Madrid, Spain; 6 Department of Electrical and Electronic Engineering, Auckland University of Technology, Auckland 1010, New Zealand; 7 Department of Electrical Engineering, United Arab Emirates University, Al Ain 15551, United Arab Emirates

**Keywords:** fractal antenna, UWB, efficiency, circular ring, WLAN, low-cost, rings

## Abstract

The present study proposes a new, highly efficient fractal antenna with ultra-wideband (UWB) characteristics. The proposed patch offers a wide simulated operating band that reaches 8.3 GHz, a simulated gain that varies between 2.47 and 7.73 dB throughout the operating range, and a high simulated efficiency that comes to 98% due to the modifications made to the antenna geometry. The modifications carried out on the antenna are composed of several stages, a circular ring extracted from a circular antenna in which four rings are integrated and, in each ring, four other rings are integrated with a reduction factor of 3/8. To further improve the adaptation of the antenna, a modification of the shape of the ground plane is carried out. In order to test the simulation results, the prototype of the suggested patch was built and tested. The measurement results validate the suggested dual ultra-wideband antenna design approach, which demonstrates good compliance with the simulation. From the measured results, the suggested antenna with a compact volume of 40 × 24.5 × 1.6 mm^3^ asserts ultra-wideband operation with a measured impedance bandwidth of 7.33 GHz. A high measured efficiency of 92% and a measured gain of 6.52 dB is also achieved. The suggested UWB can effectively cover several wireless applications such as WLAN, WiMAX, and C and X bands.

## 1. Introduction

Patch antennas have increased in popularity dramatically since the 1970s due to advances in microelectronics technology in the areas of miniaturization and electronic integration. In aeronautical, aerospace, and military contexts, antennas that are compact, lightweight, affordable, highly effective, and easy to install are critical requirements. These low-profile antennas are necessary to fulfill these needs.

In contemporary communication systems, printed antennas, which are also referred to as patch antennas or microstrip antennas, are extensively utilized. This is because commercial wireless applications face the same limitations that were once encountered in earlier times due to the proliferation of telecommunications. There exists a plethora of antenna types. In addition, wireless communication devices require more and more frequency bands due to the increasing requirements for wireless services. Since these devices are also intended to be smaller in size for real estate purposes, antennas need to reduce their size and operate in more than one frequency band while maintaining their performance.

Multiband and broadband antennas are two types of antennas that are commonly utilized in different communication systems. A multiband antenna is specifically designed to function on multiple frequency bands, making them ideal for wireless communication systems such as cellular networks. These antennas can support various frequency bands, which enables simultaneous transmission of both voice and data. Conversely, broadband antennas are designed to operate over an extensive range of frequencies, utilizing broadband elements like spiral or log-periodic antennas to cover a broad frequency range. This type of antenna is typically used in radar systems that require the detection of signals over a wide frequency range. In conclusion, multiband antennas operate over several frequency bands, while broadband antennas function across a wide frequency range. Both types of antennas offer unique advantages and are utilized in various communication and sensing systems.

An antenna for ultra-wideband (UWB) is a type of antenna that is designed to work efficiently over a wide range of frequencies, typically from a few hundred megahertz to several gigahertz. UWB antennas can come in various forms, such as monopoles, dipoles, patch antennas, and horn antennas. Some popular UWB antenna designs include the planar inverted F antenna (PIFA), the tapered slot antenna (TSA), and the printed monopole antenna.

To meet the demands of contemporary wireless communication systems, antennas with a low profile, compact size, multiband, and wideband features are greatly desired. Over the past ten years, most modern wire-free communication systems have been developed remarkably swiftly. In order to achieve high-speed broadband connections with minimal power consumption, radio networks use ultra-wideband (UWB) telecommunications technology. UWB was first designed for use with commercial radars. The two primary uses of UWB technology are in consumer electronics and wireless personal area networks (WPANs). Since its early successes in the mid-2000s, wireless UWB technology has emerged as a skill with a small number of smart structures, including the fields of medical engineering, wireless communications, and radar [1]. Owing to its inexpensive cost, reduced complexity, and increased data transfer rate, UWB technology has gained popularity since it was first commercialized. The ongoing development of UWB communication systems has led to a revolution in printed patch design methods [2,3,4], which now better match the fundamental criteria for UWB applications.

Due to its broad bandwidth, high data rate capabilities, power efficiency, interference-free transmissions, effective spectrum usage, secure communication system, and straightforward circuitry for implementation, UWB technology has attracted a lot of interest during the past 10 years [5]. The Federal Commission of Communication, situated in the United States, has designated a frequency range of 3.1 to 10.6 GHz, with a bandwidth of 7.5 GHz, for use in UWB applications [6,7,8]. Due to their simplicity and compactness, UWB antennas are a crucial component of Internet of Things (IoT) devices and wireless body area networks (WBAN) [9]. When low-cost wireless sensors are used in wearable or flexible IoT devices for continuous data transmission and low radiation power characteristics, UWB antennas find niche applications [10,11,12,13].

Recently, several approaches have been reported in the literature to obtain UWB characteristics [14,15,16,17,18,19,20,21,22,23,24,25,26,27,28]. The authors of [14] used a fractal slit in tree form to have a UWB operation. In [15], the authors suggested a compact folded patch antenna that operates over an ultra-wide bandwidth. Marzouk et al. [16] used an FR4 substrate of size 45 × 42 × 1.6 mm^3^ to construct an octagonal UWB fractal antenna. By using an RT5880 substrate and a CPW feed, Niamat et al. [17] presented a reconfigurable antenna for UWB operation. The Antipodal Vivaldi (AVA) antenna design is implemented in [18]. The authors used a fractal leaf structure inspired by ferns in nature. A planar MIMO UWB antenna with a two-port shared structure is suggested by [19]. The circular antenna has two tapered slots back-to-back. In [20], a four-element MIMO antenna for ultra-wideband (UWB) signals is proposed. A planar patch fed by a coplanar waveguide with ultra-wideband circular polarization is given in [21]. As reported in reference [22], the researchers employed a flexible UWB patch prototype with two resonators that have arc-shaped structures etched onto a PDMS substrate in their study. This design is well-suited for a range of applications that involve the human body. Fei L. et al. in [23] present a low-profile dual-band printed loop composite antenna compatible with WLAN and WiMAX systems. Tonmoy et al. in [24] used slots at the feeder and resonator to ensure UWB operation. The authors of [25] used two triangular slots and added a semi-circular tip to the patch to achieve UWB operation. The antenna reported in [26] is a circular patch fed by a coplanar waveguide (CPW). Also, in [27], a flexible patch is designed on a polyimide substrate. Also, a patch based on nanocomposite material with an impedance of 2–7 GHz and dimensions of 48 × 34.9 × 0.13 mm^3^ is proposed by [28].

In comparison to other antenna structures reported in the prior art [10,19,21,29,30,31,32], the proposed antenna has the following design objectives: to construct a new fractal antenna at a low cost; to obtain the antenna’s most compact zone; to provide UWB operation; to reach a higher peak gain and a higher radiation efficiency. The authors of [33,34,35] suggested using antennas with circular polarization (CP) to emit electromagnetic waves with an electric field that rotates in a circular pattern. This is done in order to improve signal reception and ensure better performance. The proposal from [33] suggests using a printed antenna with CP by utilizing a substrate that has a crescent shape. To achieve CP, Ref. [34] recommends the use of a square slot antenna. In [35], an inverted L-shaped CP patch was introduced by Karunesh and co-authors. For transmission and reception of data with higher data transfer rates, improved signal quality, and increased reliability, several antennas that use the MIMO technology are proposed in the literature [36,37,38].

Ultra-wideband (UWB) antennas are a type of antenna that can transmit and receive signals over a wide frequency range, typically ranging from 3.1 GHz to 10.6 GHz. UWB antennas have a number of applications in various fields, including computer science, control and systems engineering, and electrical and electronic engineering.

In computer science, UWB antennas can be used for wireless communication, particularly for high-speed data transfer between devices. UWB technology can be used for wireless USB, wireless HDMI, and other similar applications, which can eliminate the need for cables and connectors.

In control and systems engineering, UWB antennas can be used for real-time location tracking, particularly in indoor environments. UWB technology allows for very precise location tracking with an accuracy of a few centimeters, which can be used in robotics, automation, and other control and monitoring applications.

In electrical and electronic engineering, UWB antennas can be used for radar and sensing applications. UWB radar can be used for imaging, motion tracking, and other sensing applications, particularly in harsh environments where other types of sensors may not be suitable.

Overall, UWB antennas have a broad range of applications in various fields, particularly in wireless communication, real-time location tracking, and sensing applications.

The design of UWB antennas is challenging due to their requirement for high impedance bandwidth, high radiation efficiency, and low group delay distortion. It is essential to choose the appropriate antenna for the specific UWB application, based on factors such as the desired frequency range, power handling capacity, and physical size limitations. This letter is dedicated to the design and manufacture of a new UWB monopoly patch printed at cheap prices. The UWB feature is achieved by using a fractal ring shape and a partial ground plane. With a compact size, the developed patch operates over many bands with high gain and high radiation efficiency. The antenna developed in this work has a maximum gain of 7.73 dB and radiation efficiency of 98%. It is constructed on a cost-effective FR4 substrate with dimensions of 40 × 24.5 × 1.6 mm^3^. The following is an over-view of the following sections of the paper: The design process of the proposed UWB patch is described in Section 2 with the different development steps and size specifications of the proposed antenna, and the performance characteristics of the antenna are covered in Section 3, where a parametric study and a surface current distribution study are established. While the fabrication of the prototype and the discussion of the measured results are covered in Section 4. The letter is concluded in Section 5, which is followed by the references.

## 2. Antenna Design

**Figure 1:**
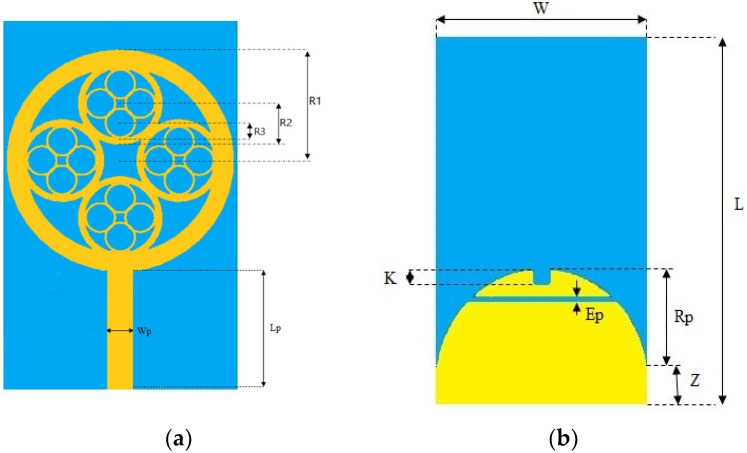
The proposed antenna structure (**a**) Top view (**b**) Back view.

The UWB fractal patch design suggested by this work, labeled with design dimensions, is exhibited on Figure 1. A low-cost FR4 substrate with a dielectric constant of 4.4 is used as the patch’s backing. The proposed fractal shape is compact in size and occupies a substrate volume of only 40 × 24.5 × 1.6 = 1568 mm^3^. A 50 Ω feed line powers a circular fractal resonator on the top face of the substrate. Additionally, to achieve good impedance matching, on the underside of the substrate, a ground plane composed of a rectangular and a half-disc-shaped part with a rectangular slot of thickness “ep” is used in place of the traditional rectangular ground plane to improve the impedance adaptation. Table 1 shows the final dimensions of the proposed UWB antenna.

### 2.1. A. Planar Fractal Antenna Planned Development Stages

Iteration 0 is composed of a circular antenna of radius 17 mm. The radius of the initiator patch is calculated for a frequency of 3.58 GHz. The radius (R) is calculated using the following equation [39,40]:

(1) $$R=\frac{F}{\sqrt{1+\left(\frac{2h}{\pi \varepsilon_{r}F}\right)\left[\ln\left(\frac{\pi F}{2h}\right)+1.7726\right]}}$$

where,

(2) $$F=\frac{8.791\times 10^{9}}{f_{r}\sqrt{\varepsilon_{r}}}$$

**Figure 2:**
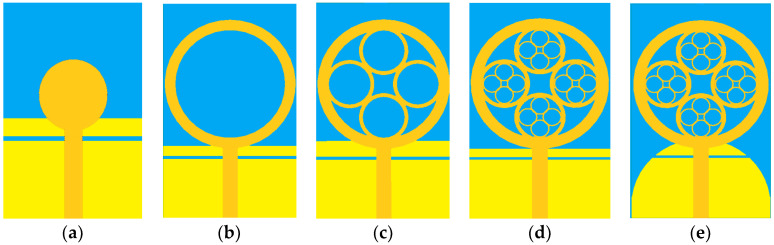
Different development stages: (**a**) initiator, (**b**) Iteration 1, (**c**) Iteration 2, (**d**) Iteration 3, (**e**) Proposed Design.

After adjusting the calculated parameters so the antenna resonates at 3.58 GHz, the required patch volume is 60 × 60 × 4 mm^3^. The evolution of the different iteration phases from the initial antenna to the proposed antenna is shown in Figure 2. Figure 2a represents the 0th iteration, it is a circular patch fed by a microstrip line. The following iteration given in Figure 2b is the 1st iteration, in which the initial structure is modified by incorporating a circular slot to improve impedance matching and increase the operating band of the patch. The ring of the first iteration is then filled with four rings of smaller radius R2 (as shown in Figure 2c). The 3rd iteration depicted in Figure 2d is obtained by incorporating sixteen rings of radius R3 to obtain a very wide operating band. Thus, in the final iteration shown in Figure 2e, which constitutes the proposed antenna, the traditional ground plane consisting of a partial rectangular ground plane is replaced by a semi-circular ground plane. The modifications proposed in Iteration 4 made it possible to obtain a UWB. At each step of the evolution of the proposed patch, four copper rings are incorporated into each ring of the previous iteration. The relations that link each ray of an iteration to the previous ones are the following: R_1_ = 12 mm, R_2_ = 3 × R_1_/8, R_3_ = 3 × R_2_/8.

### 2.2. B. The Reflection Coefficient (S11) Analysis for Various Development Stages

**Figure 3:**
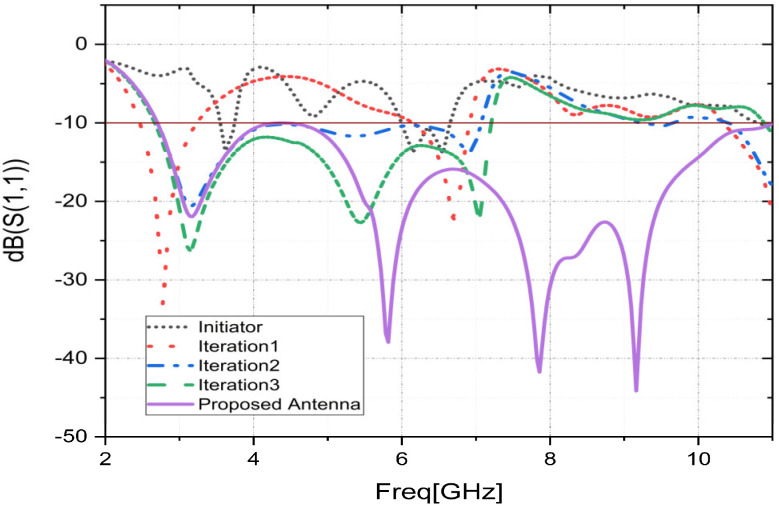
Proposed antenna reflection coefficients during various phases of development.

A fractal is a geometrical figure with a complex structure that requires the use of fractionation rules. Due to its symmetric nature, the circular antenna is used in this work. Figure 3 displays the S11 characteristics for each step-by-step design study.

The 0th iteration antenna is a simple circular shape. The initiator operates on the operating bands [3.52–3.72] and [6.00–6.60] with bandwidths of 0.2 and 0.60 GHz, respectively. The structure of the antenna has evolved in Iteration 1 to improve the results and fulfill the intended applications. The operating bands of Iteration 1 are considerably improved. However, in this stage, the antenna operates on three impedance bandwidths of 0.49, 0.74, and 0.66 GHz with a gain that reaches 4.97 dB. Thus, in the next iteration, the antenna design is modified by the introduction of four annals. The antenna of Iteration 2 has a dual-band operation with a wide bandwidth that reaches 4.35 GHz. However, the frequency band (2.70–7.05 GHz) centered on 3.14 GHz is not well suited (low loss return). Then, in the penultimate iteration, the geometry of the second iteration was modified by integrating four new rings into each ring. The new shape enables wideband operation from 2.67 to 7.18 GHz as well as a good match with a gain of up to 6.94 dB. However, the antenna presented in this iteration does not meet the X-band requirement. Thus, to further improve the antenna matching and widen the operating band to cover the Band X requirements, the patch is further evolved to Iteration 4, which gives the desired proposed UWB antenna with an increased operating bandwidth that will cover all frequency bands for the planned wireless communication applications. In the simulation of this iteration, the antenna has a UWB characteristic that covers the frequency band (2.70–11.0 GHz) with a gain that exceeds 7.7 dB. Due to this amelioration, the suggested antenna can effectively cover a variety of wireless communication applications, as listed in Table 2.

## 3. Parametric Study

This section is dedicated to the study of the effects of certain structural parameters through the HFSS simulator. The ideal dimensional specifications of the proposed patch have an impact on the performance of the antenna. The change in these parameters results in significant variations in antenna performance. The appropriate dimensions of the proposed patch can be determined using parametric studies so that it can work effectively with the best attributes.

### 3.1. Effect of Feed Line Width “Wp”

**Figure 4:**
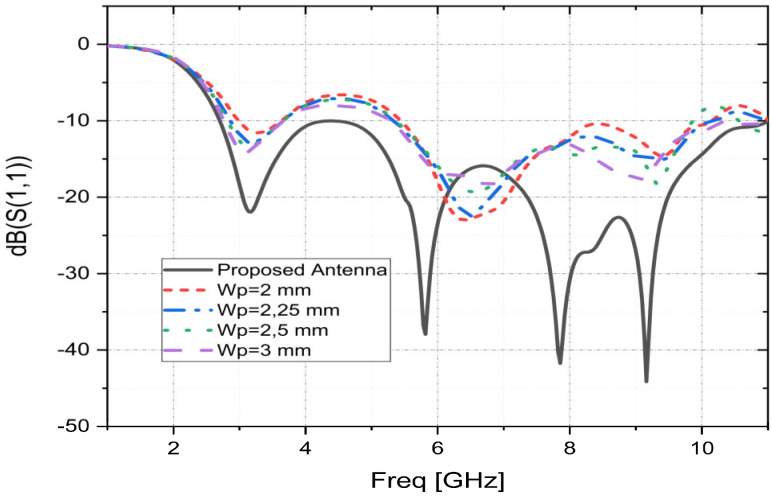
Simulated S11 parameters as functions of Wp.

The effect of the Wp on the characteristic of the reflection coefficient, keeping all other dimension parameters unchanged, is illustrated in Figure 4. Figure 4 clearly shows that the feed line width “Wp” of the proposed antenna results in a UWB operation with the best return loss characteristic (S11) and the widest operational bandwidth.

### 3.2. Effect of Ground Plane Slot Position “Pos”

**Figure 5:**
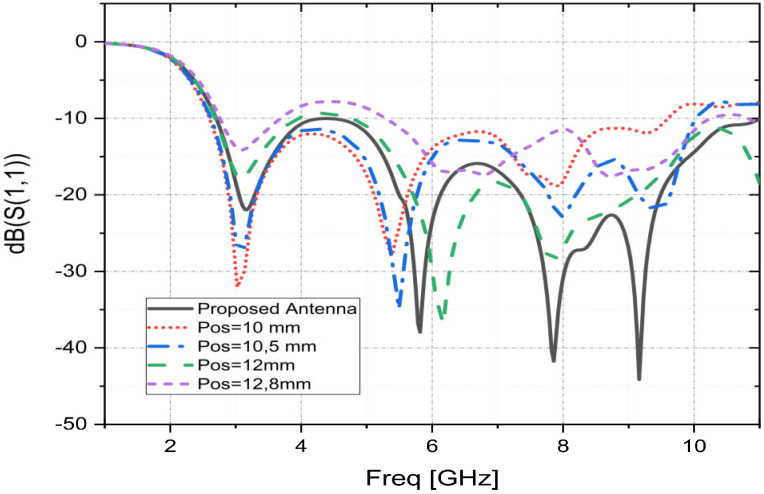
Simulated S11 parameters as functions of “Pos”.

The ground plane slot position effects on the S11 parameters of the fractal patch antenna are shown in Figure 5 while keeping all other dimensions invariant. By observing Figure 5, it is clear that varying the value of “Pos” significantly affects the impedance matching of the UWB patch and the bandwidth and that the proposed value presents the best result.

### 3.3. Effect of Ground Plane Slot Width “Ep”

**Figure 6:**
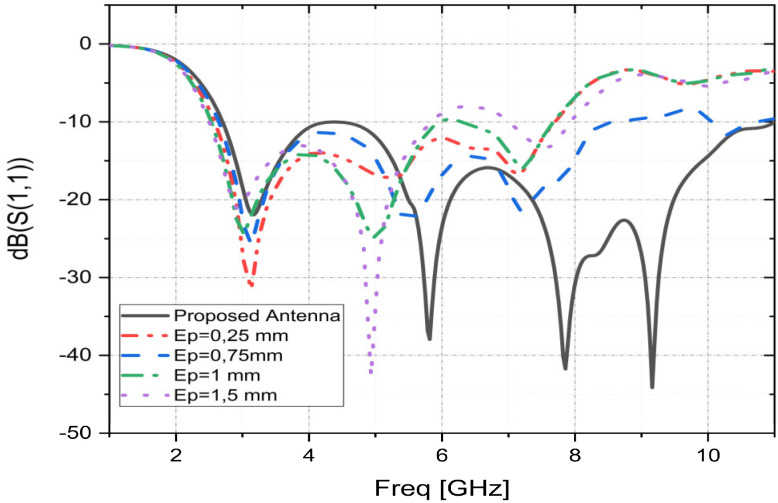
Simulated S11 parameters as functions of “Ep”.

This section examines the variations in the width “Ep” of the ground plane slot. This is another important design factor to see how it affects the reflection coefficient properties of the suggested patch. Figure 6 presents the fluctuations of the simulated reflection coefficient as a function of the variation of the slot width to better illustrate the effect of “Ep”. From Figure 6, it can be observed that the width of the slot at the ground plane “Ep” has a great influence on the operating bandwidth. The suggested width of Ep = 0.5 mm allows the proposed patch a great improvement in impedance matching and, consequently, an operational bandwidth that reaches 8.30 GHz. However, due to the variation of “Ep”, the operational bandwidth deteriorates. Given this, it can be said that the proposed patch performs optimally for Ep = 0.5 mm in terms of operational bandwidth and reflection coefficient.

### 3.4. Surface Current Distribution

**Figure 7:**
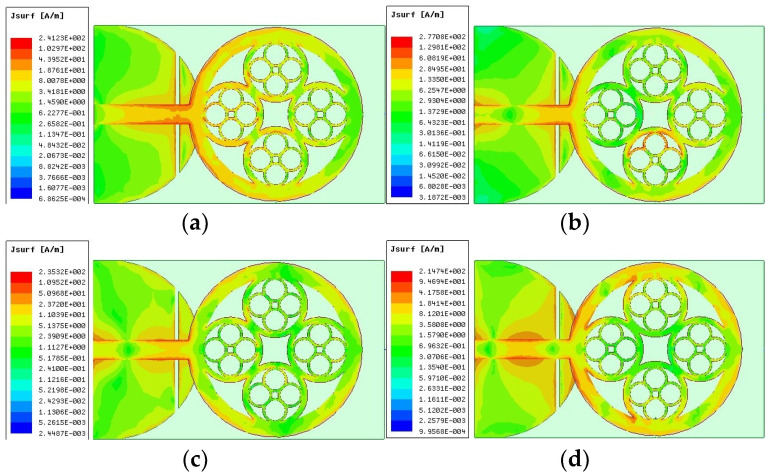
Suggested patch surface current distributions (**a**) at 3.17 GHz, (**b**) at 5.82 GHz, (**c**) at 7.86 GHz, and (**d**) at 9.16 GHz.

To properly comprehend how the UWB patch functions, Figure 7 displays the surface current distributions for the four resonant frequencies (3.17, 5.82, 7.85, and 9.16 GHz, respectively). It shows that at the upper and lower bands, the current is concentrated on the different rings, on the feeder, and on the slot at the ground plane. According to the current distribution, the rectangular ring and the strip are crucial to producing the four resonant modes of the UWB. Due to the proposed fractal shape, the resonant behavior of the patch varies with its surface current distribution. Due to the stretching of the surface currents around the rings of the fractal antenna, the fractal shape changes the distributions of the electric and magnetic fields. The way the suggested antenna disturbs the current paths raises the number of resonant frequencies. The slot at the ground plane, which has a quasi-circular shape, leads to regulated excitation and the spread of radiated waves throughout the substrate. The higher antenna bandwidth is thus explained by the interaction of the patch resonances and the additional resonances created by the new ground plane form.

## 4. Results and Discussion

**Figure 8:**
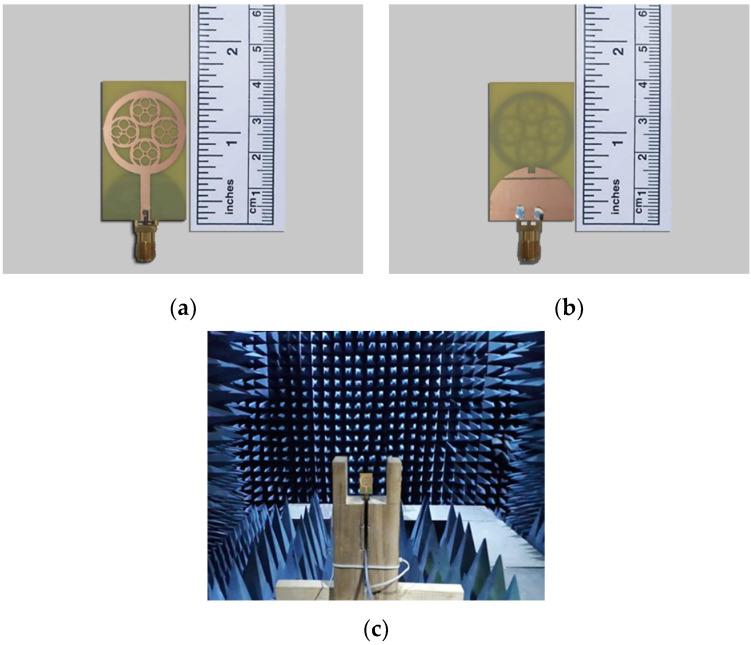
Patch prototype. (**a**) Top view, (**b**) bottom view, and (**c**) installation for measuring radiation characteristics in the anechoic chamber.

HFSS software was used for antenna design and optimization. After that process, the suggested optimal patch is manufactured and measured to confirm the simulated results. The front and back views of the prototype, as well as the measurement setup in the anechoic chamber, are depicted in Figure 8a–c.

**Figure 9:**
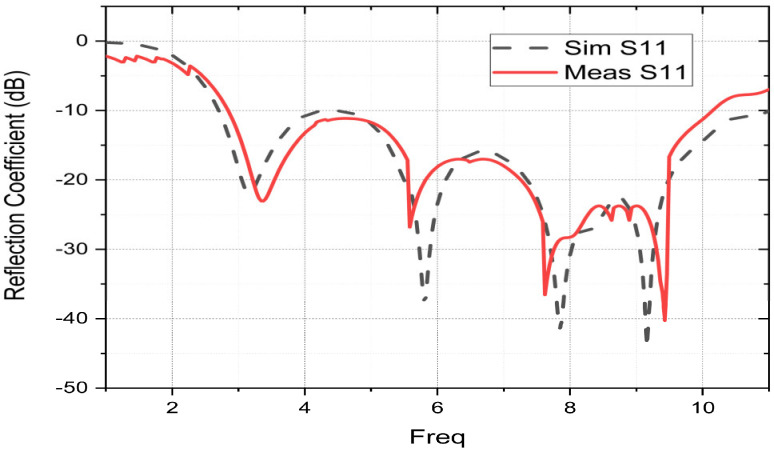
Comparison of measured and simulated results of the proposed fractal patch.

The S11 of the prototype patch is measured with the use of the vector network analyzer. Figure 9 compares the S11 parameters measured and simulated using the HFSS software. The results from simulation and measurement are in good agreement, and they both support the UWB operating properties with four resonance frequencies. An improvement in input impedance between 4.0 and 4.8 GHz is noted in the empirical results in comparison to the simulated ones. Moreover, a slight decrease in the operating band is observed for the measured results. This drop could be caused by the impact of the high frequency on the FR4, manufacturing tolerance, simulation frequency width, measurement circumstances, the dielectric permittivity in the substrate, or the SMA connector’s soldering conditions. According to the obtained measurement results, the UWB operation is affirmed. The manufactured prototype can cover the operating band [2.83–10.16] GHz with a measured bandwidth of 7.33 GHz. The results of S11 obtained by the measurement show that the suggested fractal patch has a good operation with reliable performance. The suggested antenna accommodates the bandwidth needs of several wireless protocols, such as WLAN, Wi-MAX, Wi-Fi, ITU assigned all C band, receive frequency, and radiolocation.

**Figure 10:**
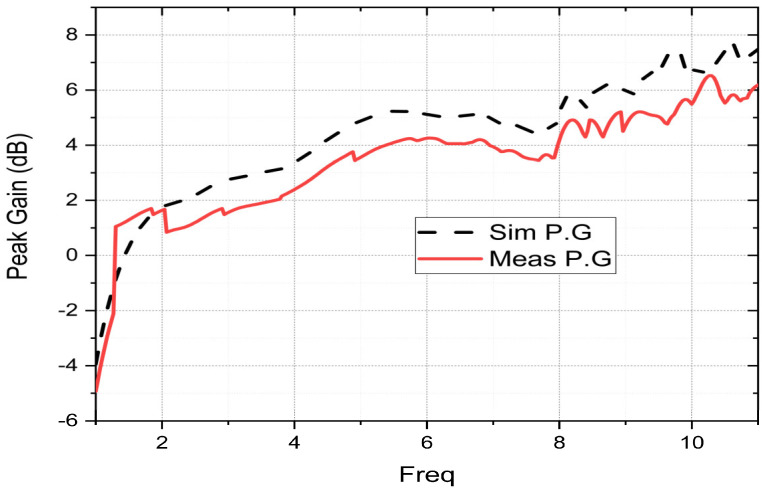
Peak gain of the suggested patch.

**Figure 11:**
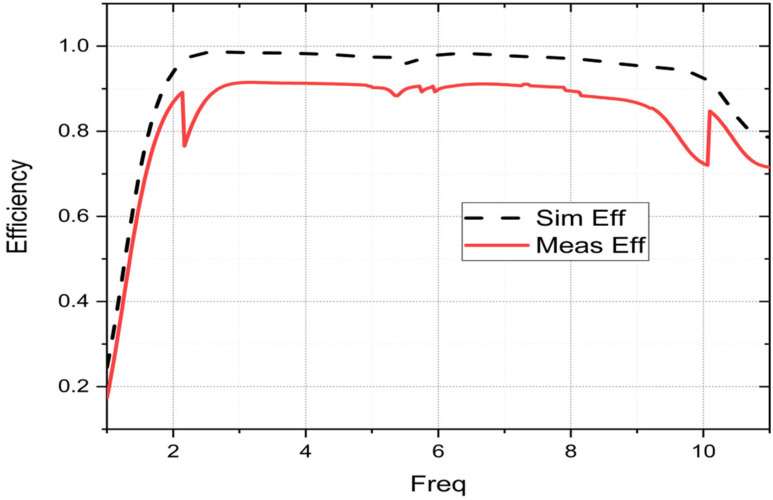
Radiation efficiency of the suggested patch.

The radiation properties study of the suggested patch in terms of peak gain, radiation efficiency, and the radiation pattern is presented in this section. The measurement results are used to confirm the simulation results. The radiation parameters of the patch are realized in an anechoic chamber, as shown in Figure 8c. Both Figure 10 and Figure 11 show the peak gain and radiation efficiency results of the fractal antenna. The results of the simulation and measurement can be seen to differ slightly. Compared to the simulated pattern, the measured peak gain pattern is smaller. This difference may be due to the lower matching level of the manufactured patch than the simulated patch; this mismatch may be due to manual soldering, the effect of the connector, or impurities in the substrate used for fabrication. Furthermore, it is clear from Figure 10 that as the frequency increases, the peak gain also increases. This could be explained by the fact that the size of the patch becomes larger than its wavelength when the frequency increases. It may be up to 6.52 dB. However, as shown in Figure 11, the measured radiation efficiency reaches a value of 91%. The effectiveness of an antenna in directing or capturing signals in a particular direction, compared to an isotropic radiator, is referred to as its gain. Typically, the gain of an antenna increases as the signal frequency increases up to a certain threshold. However, at higher frequencies, the gain starts to decrease due to several factors, including losses in the antenna and the growing difficulty of efficiently directing or capturing the signal. In contrast, radiation efficiency presents the ability of an antenna to convert the electrical energy it receives into electromagnetic radiation that can be transmitted through the air. The radiation efficiency of an antenna also varies with frequency but in the opposite direction to its gain. With an increase in frequency, the radiation efficiency tends to decrease due to increased resistive losses and decreased coupling between the antenna and the surrounding environment. As a result, as the frequency increases, the gain of the antenna will increase to a maximum, while the radiation efficiency will decrease at those frequencies.

**Figure 12:**
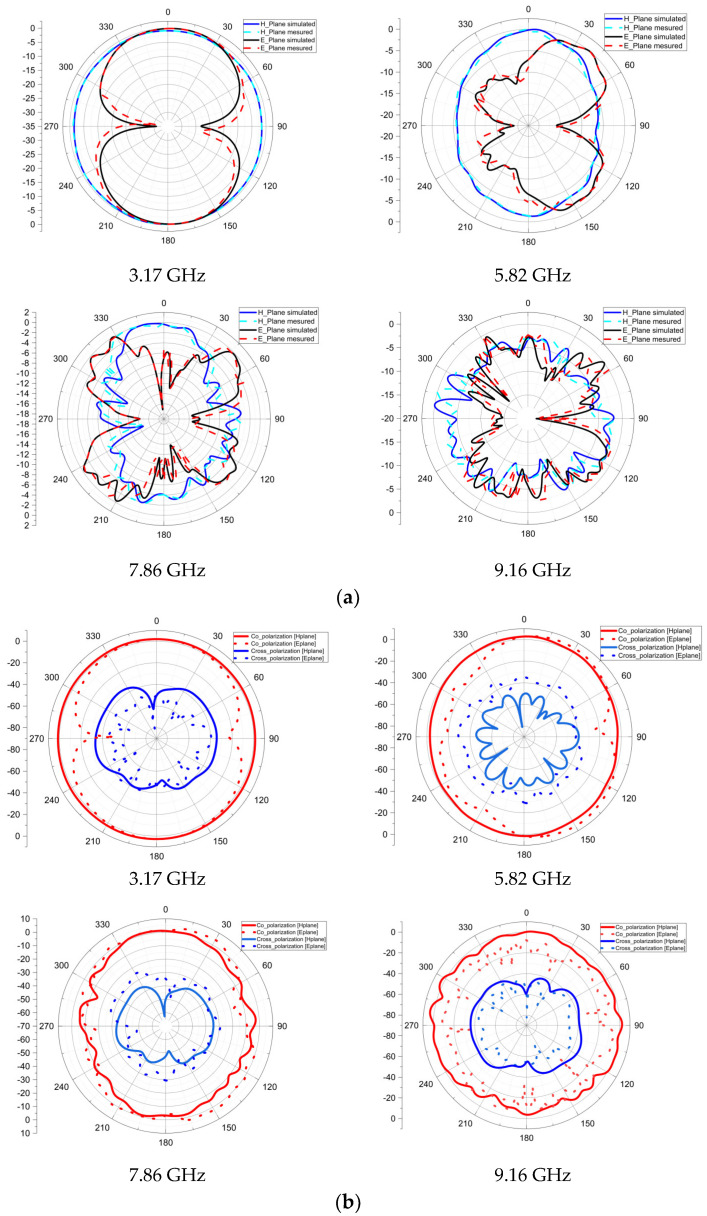
(**a**) 2D radiation pattern and (**b**) Co-polarization and cross-polarization.

The simulated and measured 2D radiation patterns in the E and H planes at 3.17, 5.82, 7.86, and 9.16 GHz are displayed in Figure 12a. The measured and simulated results display excellent agreement, as seen in Figure 12a. Effectively radiating across the operating band is the prototype patch. In the first two resonant frequencies, the radiation pattern in the H-plane is omnidirectional and quasi-omnidirectional in the high frequencies. In contrast, in the E-plane, the radiation pattern is bidirectional in the first two resonant frequencies and quasi-omnidirectional in the other two frequencies. The influence of the high frequencies on the FR-4 substrate is the cause of the modification of the radiation pattern observed at high frequencies. Co-polarization and cross-polarization are terms used in the field of radio communications and electromagnetic waves to describe the relationship between transmitting and receiving antennas. The former aims to maximize signal strength and minimize interference, while the latter aims to reduce the interference caused by reflections. Both are important considerations when designing a communication system. Figure 12b illustrates co-polarization and cross-polarization.

Group delay and time domain characteristics are two different measures of an antenna’s performance in the time domain.

The analysis of the behavior of an antenna in the time domain is known as the antenna time domain study, and it is crucial to comprehend the transient and dynamic behavior of an antenna [36,37,38]. This type of analysis can reveal important information about the antenna’s radiation pattern, polarization, bandwidth, and impedance.

The study of the antenna time domain plays a critical role in understanding how an antenna behaves when transmitting or receiving signals that change over time. By examining the antenna’s time–domain behavior, engineers can tailor its design to meet specific requirements, such as radar, wireless communication, or satellite communication.

Furthermore, time–domain analysis can help detect potential issues with the antenna’s performance, such as impedance mismatches, noise, or interference. This knowledge can then be used to enhance the antenna’s design and performance, resulting in improved signal quality, increased efficiency, and reduced interference.

In summary, studying the antenna time domain is a vital component of antenna engineering, as it provides valuable insights into the antenna’s behavior, leading to optimized design and performance for specific applications.

**Figure 13:**
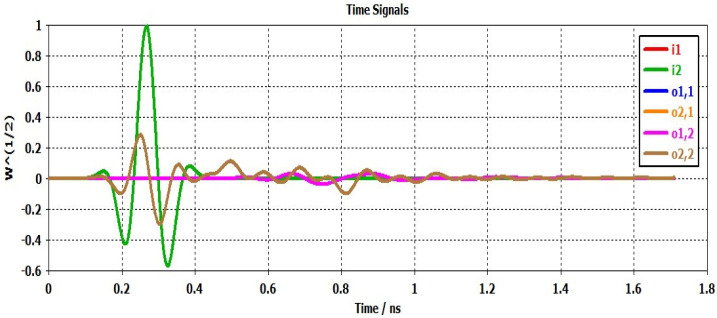
Time–domain response.

In order to demonstrate the time–domain performance of the antenna, a pair of identical antennas are positioned in front of each other, with one serving as the transmitter and the other as the receiver, and their faces directed towards each other. These antennas are positioned at a distance of five times the wavelength of the lowest operating frequency to establish a far-field environment. The time domain response of the proposed antenna is depicted in Figure 13. Where i1 indicates the input signal of port 1 of the first antenna, o1 is the output signal of port 1 of the first antenna, i11 is the input signal of port 1 of the first and second antennas, and i12 is the input signal of port 1 of the first antenna and port 2 of the second antenna.

**Figure 14:**
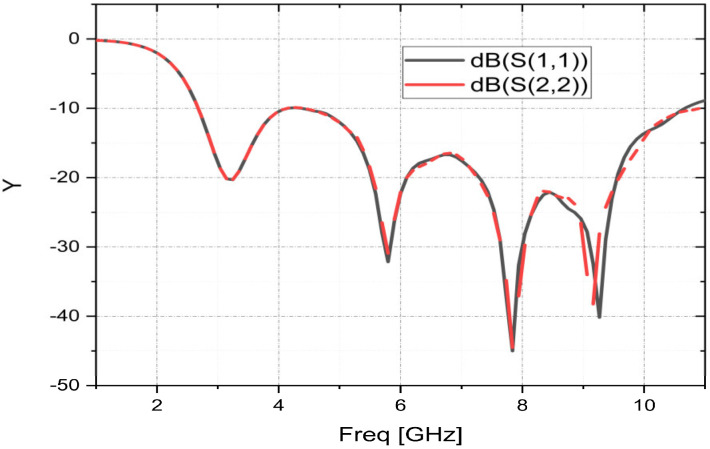
Group delay.

In other words, the time–domain characteristics describe how an antenna reacts to changes in the input signals over time, while the group delay describes how the antenna affects the phase of the different frequency components of the signal as they pass through it. Group delay is depicted in Figure 14.

Table 3 presents an assessment of the recommended UWB patch antenna as compared to patch configurations described in the existing literature based on antenna size, substrate type, operational bandwidth, and peak gain. The antenna highlighted in reference [10] is relatively large in size and offers a gain that does not exceed 3.78 dB with two narrow bandwidths. Although the antennas proposed in references [19,21] offer wide bandwidth, they have large dimensions that may pose challenges related to space requirements. Despite their compact physical size, the antennas mentioned in [33,34,35] have a complex design and exhibit low gain and low bandwidth. The patch antennas suggested in other references mentioned in the table are characterized by large sizes and low gain. In contrast, the patch antenna proposed in this article exhibits a clear competitive edge over prior research. By utilizing an FR4 substrate, this antenna can be manufactured at a low cost while still achieving a small form factor of 40 × 24.5 × 1.6 mm^3^, an impedance bandwidth of 8.3 GHz, and a high gain of 6.25 dB.

## 5. Conclusions

In this paper, a UWB fractal monopole patch is designed and analyzed for wireless communication applications. With the aid of HFSS software, the proposed antenna’s structure was designed and examined. The developed antenna only needs a tiny area of 40 × 24.5 × 1.6 mm^3^. A fractal ring resonator and a ground plane made up of a rectangular part and a half-disk part with a rectangular slot are used to create the UWB operation. The manufactured prototype’s measured results and simulated results match up reasonably well. The proposed antenna operates at measured bandwidths of 7.33 GHz (2.83–10.16 GHz). Additionally, it reports a maximum measured radiation efficiency of roughly 92% and a measured peak gain of 6.52 dB. The developed patch is lightweight, small in size, and has good radiation parameters that enable it to compete in a variety of wireless communication applications, including WLAN, Wi-MAX, Wi-Fi, ITU, C band, and radiolocation, among others.

## Figures and Tables

**Table 1:** Size specifications for the proposed antenna.

Parameters	L	W	Rp	Ep	Z	R1	R2	R3	Lp	Wp	K
**Values (mm)**	40	24.5	12.25	0.5	4	12	4.5	1.68	13.08	2.75	2

**Table 2:** Frequency bands that the suggested antenna would cover.

Bandwidth [GHz]	Covered Commercial Bands
**[2.70–11.0]**	5.15–5.825 GHz WLAN; 3.4–3.69 GHz, 5.25–5.85 GHz Wi−MAX; 3.6575–3.690 GHz, 5.180–5.825 GHz Wi−Fi; ITU assigned all C band transmit frequency (5.85–6.425 GHz,6.425–6.725 GHz, 6.725–7.025 GHz, 5.975–6.475 GHz, 5.725–6.025 GHz), and receive frequency (3.625–4.200 GHz, 3.400–3.625 GHz, 3.650–4.150 GHz, 3.7–4.0 GHz) around the world, and ITU assigned amateur radio (10–10.50 GHz) and amateur satellite (10.45–10.50 GHz) applications in the X band.

**Table 3:** Comparative evaluation of the suggested antenna against different published patches in the literature.

Ref No	Electrical Size	Subs Type	Band Operational (GHz)	Resonant Frequency (GHz)	Peak Gain (dB)
[10]	0.189 **λ** × 0.189 **λ**	FR4	[2.37–3.78], [5.15–5.85]	2.65, 3.45, 5.65	1.62 to 3.1 1.74 to 3.78
[19]	110 × 120 (3.66)	Rogers RO4350B	[3.00–10.00]	3.00, 6.00	6.00
[21]	75 × 63 (3.2)	Rogers RO4232	[3.10–10.6]	2.4, 3.2	3.50
[29]	0.22 × 0.22	FR4	[2.3–2.6], [3.3–3.7]	2.46, 3.5	2.61, 2.7
[30]	0.2 **λ** × 0.13 **λ**	FR4	[2.24–2.5], [3.6–3.99], [4.4–4.6], [5.71–5.9]	2.43, 3.83, 4.48, 5.8	2.2, 2.8, 3.3, 4.2
[32]	0.28 **λ** × 0.14 **λ**	FR4	[2.2–3.4], [3.34–4.52]	Not specified	2.2 to 2.4
[33]	0.22 **λ** × 0.22 **λ**	FR4	[4.80–5.99]	5.5	2.5
[34]	0.148 **λ** × 0.161 **λ**	FR4	[4.65–6.72]	5.2	Not specified
[35]	0.176 **λ** × 0.176 **λ**	FR4	[3.48–5.86]	5.1	Not specified
**This work**	0.171 **λ** × 0.104 **λ**	**FR4**	**[2.70–11.0]**	**3.17, 5.82, 7.86, 9.16**	**1.7 to 6.25**

## References

[1] Kirtania S.G., Younes B.A., Hossain A.R., Karacolak T., Sekhar P.K. (2021). CPW-Fed Flexible Ultra-Wideband Antenna for IoT Applications. Micromachines.

[2] Kikuta K., Hirose A. (2015). Compact Folded-Fin Tapered Slot Antenna for UWB Applications. IEEE Antennas Wirel. Propag. Lett..

[3] Zhong Y.-W., Yang G.-M., Zheng L.-R. (2014). Planar circular patch with elliptical slot antenna for ultrawideband communication applications. Microw. Opt. Technol. Lett..

[4] Ojaroudi M., Ghobadi C., Nourinia J. (2009). Small Square Monopole Antenna with Inverted T-Shaped Notch in the Ground Plane for UWB Application. IEEE Antennas Wirel. Propag. Lett..

[5] Hirt W. (2003). Ultra-wideband radio technology: Overview and future research. Comput. Commun..

[6] Alam J., Faruque M.R.I., Hasan M., Islam M.T. (2017). Split quadrilateral miniaturised multiband microstrip patch antenna design for modern communication system. IET Microw. Antennas Propag..

[7] Ramos A., Lazaro A., Girbau D. (2016). RFID and Wireless Sensors Using Ultra-Wideband Technology.

[8] Anveshkumar N., Gandhi A.S. (2018). Lumped Equivalent Models of Narrowband Antennas and Isolation Enhancement in a Three Antennas System. Radioengineering.

[9] Davoli L., Belli L., Cilfone A., Ferrari G. (2017). From Micro to Macro IoT: Challenges and Solutions in the Integration of IEEE 802.15.4/802.11 and Sub-GHz Technologies. IEEE Internet Things J..

[10] Dumoulin A., John M., Ammann M.J., McEvoy P. (2012). Optimized Monopole and Dipole Antennas for UWB Asset Tag Location Systems. IEEE Trans. Antennas Propag..

[11] Gao G.-P., Hu B., Wang S.-F., Yang C. (2018). Wearable Circular Ring Slot Antenna With EBG Structure for Wireless Body Area Network. IEEE Antennas Wirel. Propag. Lett..

[12] Islam M.T., Islam M., Samsuzzaman M., Faruque M.R.I., Misran N. (2015). A Negative Index Metamaterial-Inspired UWB Antenna with an Integration of Complementary SRR and CLS Unit Cells for Microwave Imaging Sensor Applications. Sensors.

[13] Kang C.-H., Wu S.-J., Tarng J.-H. (2011). A Novel Folded UWB Antenna for Wireless Body Area Network. IEEE Trans. Antennas Propag..

[14] Nejdi I.H., Das S., Rhazi Y., Madhav B.T.P., Bri S., Aitlafkih M. (2022). A Compact Planar Multi-Resonant Multi-Broadband Fractal Monopole Antenna for Wi-Fi, WLAN, Wi-MAX, Bluetooth, LTE, S, C, and X Band Wireless Communication Systems. J. Circuits Syst. Comput..

[15] Qu S.-W., Ruan C.-L., Xue Q. (2007). A Planar Folded Ultrawideband Antenna With Gap-Loading. IEEE Trans. Antennas Propag..

[16] Marzouk M., Nejdi I.H., Rhazi Y., Saih M. Multiband and Wide Band Octagonal Fractal Antenna for Telecommunication Applications. Proceedings of the 2022 8th International Conference on Optimization and Applications (ICOA).

[17] Hussain N., Awan W.A., Naqvi S.I., Ghaffar A., Zaidi A., Iftikhar A., Li X.J. (2020). A Compact Flexible Frequency Reconfigurable Antenna for Heterogeneous Applications. IEEE Access.

[18] Biswas B., Ghatak R., Poddar D.R. (2017). A Fern Fractal Leaf Inspired Wideband Antipodal Vivaldi Antenna for Microwave Imaging System. IEEE Trans. Antennas Propag..

[19] Nie L.Y., Lin X.Q., Yang Z.Q., Zhang J., Wang B. (2018). Structure-Shared Planar UWB MIMO Antenna With High Isolation for Mobile Platform. IEEE Trans. Antennas Propag..

[20] Tang Z., Wu X., Zhan J., Hu S., Xi Z., Liu Y. (2019). Compact UWB-MIMO Antenna with High Isolation and Triple Band-Notched Characteristics. IEEE Access.

[21] Das S., Islam H., Bose T., Gupta N. (2019). Ultra Wide Band CPW-Fed Circularly Polarized Microstrip Antenna for Wearable Applications. Wirel. Pers. Commun..

[22] Simorangkir R.B., Kiourti A., Esselle K.P. (2018). UWB Wearable Antenna with a Full Ground Plane Based on PDMS-Embedded Conductive Fabric. IEEE Antennas Wirel. Propag. Lett..

[23] Liu F., Xu K., Zhao P., Dong L., Wang G. (2017). Uniplanar dual-band printed compound loop antenna for WLAN/WiMAX applications. Electron. Lett..

[24] Saha T.K., Goodbody C., Karacolak T., Sekhar P.K. (2018). A compact monopole antenna for ultra-wideband applications. Microw. Opt. Technol. Lett..

[25] Ali E.M., Awan W.A., Alizaidi M.S., Alzahrani A., Elkamchouchi D.H., Falcone F., Ghoneim S.S.M. (2023). A Shorted Stub Loaded UWB Flexible Antenna for Small IoT Devices. Sensors.

[26] Zahran S.R., Abdalla M.A., Gaafar A. (2018). Time domain analysis for foldable thin UWB monopole antenna. AEU Int. J. Electron. Commun..

[27] Wang Z., Qin L., Chen Q., Yang W., Qu H. (2019). Flexible UWB antenna fabricated on polyimide substrate by surface modification and in situ self-metallization technique. Microelectron. Eng..

[28] Hamouda Z., Wojkiewicz J., Pud A.A., Kone L., Bergheul S., Lasri T. (2018). Flexible UWB organic antenna for wearable technologies application. IET Microw. Antennas Propag..

[29] Ali W.A.E., Ashraf M.I., Salamin M.A. (2020). A dual-mode double-sided 4 × 4 MIMO slot antenna with distinct isolation for WLAN/WiMAX applications. Microsyst. Technol..

[30] Chouhan S., Panda D.K., Kushwah V.S., Singhal S. (2019). Spider-shaped fractal MIMO antenna for WLAN/WiMAX/Wi-Fi/Bluetooth/C-band applications. AEU Int. J. Electron. Commun..

[31] Ben Nsir C., Ribero J.-M., Boussetta C., Gharsallah A. A Wide Band Transparent Koch Snowflake Fractal Antenna Design for Telecommunication Applications. Proceedings of the 2019 IEEE 19th Mediterranean Microwave Symposium (MMS).

[32] Choukiker Y.K., Behera S.K. (2017). Wideband frequency reconfigurable Koch snowflake fractal antenna. IET Microw. Antennas Propag..

[33] Kulkarni J., Sim C.-Y., Poddar A.K., Rohde U.L., Alharbi A.G. (2022). A Compact circularly polarized rotated L-shaped antenna with J-shaped defected ground strucutre for wlan and V2X applications. Prog. Electromagn. Res. Lett..

[34] Midya M., Bhattacharjee S., Mitra M. (2018). Compact cpw-fed circularly polarized antenna for wlan application. Prog. Electromagn. Res. M.

[35] Srivastava K., Mishra B., Singh R. (2021). Microstrip-line-fed inverted L-shaped circularly polarized antenna for C-band applications. Int. J. Microw. Wirel. Technol..

[36] Desai A., Kulkarni J., Kamruzzaman M.M., Hubalovsky S., Hsu H.-T., Ibrahim A.A. (2022). Interconnected CPW Fed Flexible 4-Port MIMO Antenna for UWB, X, and Ku Band Applications. IEEE Access.

[37] Khangarot S., Sravan B., Aluru N., Saadh A.M., Poonkuzhali R., Kumar O.P., Ali T., Pai M.M. (2020). A compact wideband antenna with detailed time domain analysis for wireless applications. Ain Shams Eng. J..

[38] Addepalli T., Desai A., Elfergani I., Anveshkumar N., Kulkarni J., Zebiri C., Rodriguez J., Abd-Alhameed R. (2021). 8-Port Semi-Circular Arc MIMO Antenna with an Inverted L-Strip Loaded Connected Ground for UWB Applications. Electronics.

[39] Journals I., Devi R., Neog D.K. (2015). Determination of Radius of Circular Microstrip Antenna Using Clonal Selection Algorithm. IOSR J. Electron. Commun. Eng. Ver. I.

[40] (2015). Antenna Theory: Analysis and Design.

